# Bone health: Quality versus quantity

**DOI:** 10.1016/j.jposna.2024.100054

**Published:** 2024-04-07

**Authors:** Anxhela Docaj, Alessandra Carriero

**Affiliations:** Department of Biomedical Engineering, The City College of New York, New York, NY, USA

**Keywords:** Bone, Quality, Quantity, Health, Fragility

## Abstract

Healthy bone has the ability to resist deformation and fracture while adapting to applied mechanical loads. These properties of bone depend on characteristics of its extracellular matrix. This review focuses on the contribution of bone quality and quantity to bone health and highlights current and promising future clinical approaches to measure bone health in the pediatric population. Bone’s unique material properties are derived from its highly organized, hierarchical composite structure, together with its modeling and remodeling dynamics and microdamage mechanisms. Pediatric bone diseases and disorders affect the biological processes that regulate its quality, negatively impacting the extracellular matrix and causing bone fragility. Laboratory bone analysis from human biopsies or animal models of human bone diseases allows high detail examination of the mechanisms contributing to bone fragility. Conversely, clinical measurements of bone fragility are difficult and limited due to the inaccessibility of the material. Because bone quality directly affects fracture resistance, both structure and composition should be used in fracture risk calculation rather than bone mineral density or bone quantity alone. Thus, to advance clinical evaluation of bone fragility, future studies are needed to determine which characteristics of bone quality can be applied to clinical practice to predict bone fragility. New and effective clinical tools are needed to predict fracture risk taking bone quality into consideration.

**Key Concepts:**

(1) Bone quality and bone quantity are both fundamental for resistance to deformity and fracture.
(2) Pediatric bone diseases and disorders alter bone’s composition and structure, compromising bone quality and increasing vulnerability to fracture.
(3) Current clinical approaches to assess bone fragility and fracture risk rely mainly on bone quantity measurements from DEXA scans.
(4) DEXA bone mineral density poorly correlates with bone’s resistance to fracture, both in adults and children.
(5) Future clinical approaches to measure bone health should account for bone quality in order to predict fracture risk.

## Introduction

**Figure 1:**
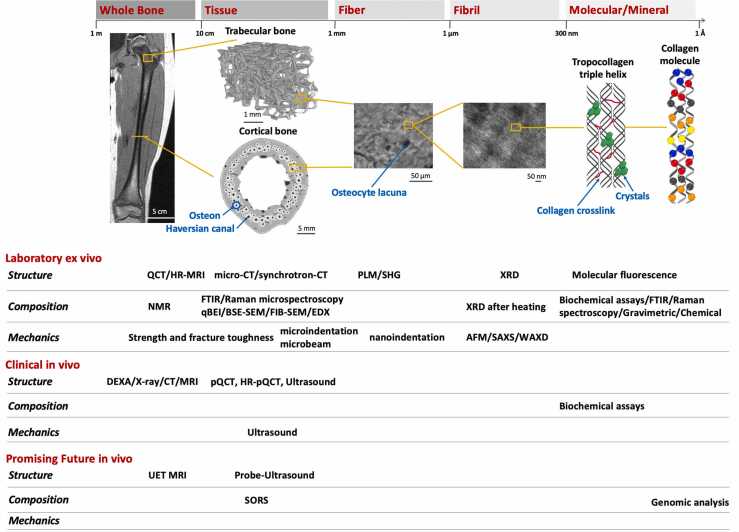
The hierarchical structure of bone from macro- to nanoscale. For specific operating length scales, there are reported the techniques that provide structural, compositional and mechanical properties of bone, either currently used in laboratories, clinically or that are promising for future clinical use. MRI femur image with permission from Carriero et al., 2009 [3]. Fibril picture with permission from Klosowski et al., 2016 [4]. AFM, atomic force microscopy; BSF-SEM, backscattered electron scanning electron microscopy; CT, computed tomography; DEXA, dual-energy x-ray absorptiometry; EDX, energy-dispersive x-ray analysis; FIB-SEM, focused ion beam SEM; FTIR, fourier transform infrared; HR-MRI, high-resolution magnetic resonance imaging; HR-pQCT, high-resolution peripheral quantitative CT; NMR, nuclear magnetic resonance imaging; pQCT, peripheral quantitative CT; qBEI, quantitative backscattered electron imaging; QCT, quantitative CT; SAXS, small-angle x-ray scattering; SHG, second harmonic generation microscopy; SORS, spatially offset Raman spectroscopy; UET MRI, ultrashort echo time MRI; WAXD, wide-angle x-ray diffraction; XRD, x-ray diffraction analysis.

Healthy bone is a dynamic living tissue with remarkable mechanical properties and the ability to adapt to applied loads. Bone is both strong and tough, as it resists deformations and fracture, respectively [1], [2]. Bone derives these exceptional mechanical properties from its unique composite nature. It is made primarily of mineral crystals embedded in an organic component, with an organized hierarchical structure from the atomic to macroscopic scale (Fig. 1). Because of its composite nature, bone benefits from the properties of its phase constituents both mineral and organic [1], [2]. Maintaining the structural integrity of bone is therefore very clinically important to preserve its function and adaptability, particularly during skeletal growth. In children, diseases and abnormal loading conditions on the skeleton that occur secondary to other disorders, such as cerebral palsy, may result in osteopenia, compromising bone quantity, or may alter composition and structure, compromising bone quality. Changes to both bone quantity and quality increase bone’s vulnerability to deformity and fragility. This article focuses on the contribution of bone quality and quantity to bone health and highlights current and promising future clinical approaches to measure bone health in the pediatric population.

### Bone quantity

Traditionally, bone quantity has been considered the predictor of fracture risk. Specifically, low bone mass or low bone mineral density (BMD; equivalent to the amount of bone mineral per unit cross-sectional area) has been associated with an increased fracture rate observed with aging and bone disease in adults. However, in the past three decades, studies have demonstrated that bone quantity cannot be the sole factor responsible for increased fracture rate, nor can bone mass alone explain benefits of drug therapies for bone fragility in adults and children [5], [6], [7], [8], [9], [10]. Therefore, there has been increased interest in factors regulating bone quality, such as composition, structure, micro-damage mechanisms, and modeling and remodeling processes.

### Bone quality

Bone is a composite material made primarily of collagen type I, a fibrous organic protein constituting the backbone of the extracellular matrix, surrounded by hydroxyapatite crystals. The extracellular matrix is made of an organic component, with approximately 90% collagen, 5% non-collagenous proteins (NPCs), 2% glycoproteins, and by an inorganic component composed of mineral, and water [11]. Although small in content, the NCPs and glycoproteins, including osteopontin and osteocalcin, are key players in bone formation, mineralization, and regulation of bone formation/breakdown [11], [12]. Type I collagen is a triple helical molecule containing 2 symmetric α_1_ amino acid chains and one α_2_ amino-acid chain, synthesized respectively by the *COL1A1* and *COL1A2* genes [13]. Collagen in bone is mostly mineralized by very small crystals of carbonated hydroxyapatite, an impure version of calcium phosphate, Ca_10_(PO_4_)_6_(OH)_2_ [14]. It has a variety of impurities, mainly carbonate replacing the phosphate groups (around 4–6%), but also lower quantities of magnesium, fluoride, and sodium [15]. The shape, distribution, and composition of the mineral crystals embedded in the bone have a direct impact on its mechanical behavior [16], [17], [18].

**Figure 2:**
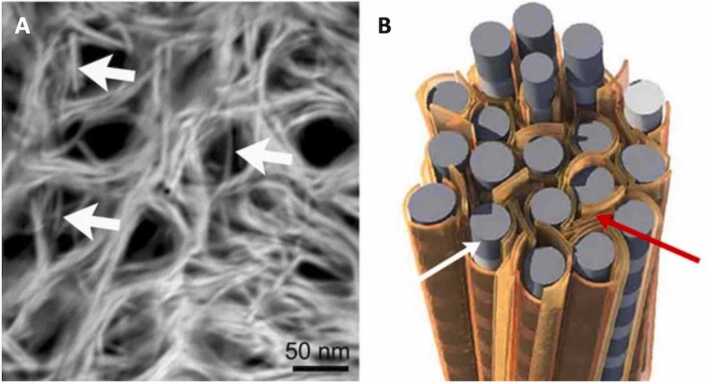
(A) Stacks of mineral lamellae (thin polycrystalline plates, otherwise referred to as platelets) wrap around circular dark “holes” of collagen fibrils as seen in transmission electron microscopy (TEM) images of transverse cortical femur cross-section of a healthy 19-year-old male. Single mineral lamellae passing through collagen fibrils are marked with white arrows. (B) Schematic of mineral lamellae platelets (orange) surrounding fibrils (gray) as marked by the white arrow. Red arrow shows mineral lamellae sheets that are stacked between adjacent collagen fibrils. Adapted with permission from Grandfield et al., 2018 [21].

Under normal conditions, these bone apatite crystals follow an orderly arrangement within the collagen framework [19]. The mineralized collagen molecules organize into fibrils (Fig. 1). The apatite crystals aggregate into elongated mineral nanoplatelets that arrange periodically in the intrafibrillar gaps following the direction of the fibril, [20] and wrap around collagen fibrils on the extrafibrillar surfaces (Fig. 2) [21]. The collagen components connect to each other through enzymatic crosslinking that provides support to the mineral phase, and stability and elasticity to the bone structure (Fig. 1) [15]. Fibrils then assemble into fibers at the tissue level, which organize into layers called lamellae. In cortical bone, lamellae organize concentrically around the Haversian canals, bone’s major blood vessels running longitudinally, to form osteons, bordered by a hypermineralized tissue layer called bone cement (Fig. 1). Cortical bone also has Volkmann canals, which help deliver blood and nutrients to the bone. Both trabecular and cortical bone have osteocyte lacunae (little caves) where bone cells are found and connect to each other (Fig. 1).

### Bone strength and toughness in healthy and diseased pediatric populations

**Figure 3:**
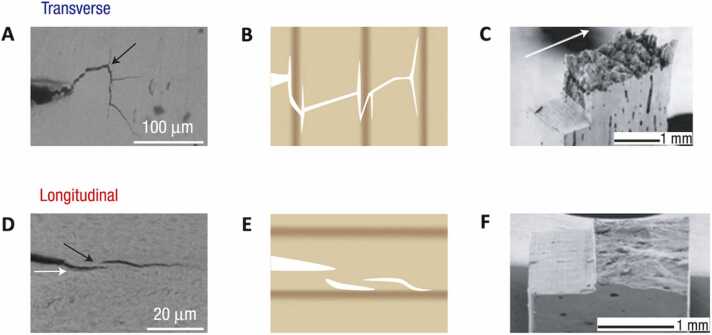
Crack profiles, schematic diagrams and environmental SEM fractography images of human cortical bone in the transverse and longitudinal orientations show that bone is more difficult to break than to split. In the transverse (‘breaking’) direction (A-C), the crack path is (A) tortuous with (B) many deflections at the cement lines and (C) through-thickness twists which lead to a very rough fracture surface. In the longitudinal (‘splitting’) direction (D-F), the crack trajectory is (D) straight and much smoother with (E) no visible deflections at the cement sheaths but instead following them leading to (F) a relatively flat fracture surface. Adapted with permission from Koester et al., 2008 [35]. SEM, scanning electron microscopy.

Bone’s complex hierarchical structure and composition are the foundation of its mechanical properties. Bone strength (i.e., resistance to deformation) depends on both bone quantity and quality [22], [23], [24], [25], [26]. Conversely, bone toughness (i.e.*,* resistance to fracture) depends solely on properties of bone quality, such as bone micro-architecture, collagen fiber organization and mineralization at the tissue level, as well as collagen-mineral interaction, structure and organization at the sub-cellular level [27], [28], [29], [30], [31], [32], [33], [34], [35], [36], [37]. At the nanoscale level, mineralized fibrils confer strength and stiffness to bone, and their arrangement in lamellae and osteons at the micro-scale level allows the distribution of applied loading forces, enabling bone to maintain its mechanical integrity [38]. At cement lines, microcracks formation and crack deflections at osteonal interfaces dissipate energy and increase resistance to fracture while bone is sustaining loads. As a result, the osteonal alignment along the long axis of the bone makes it 5 times more resistant to break than to split (Fig. 3) [39]. Disease and pathological conditions in children alter bone composition, disrupt its hierarchical structure (Fig. 1), and change the impact of loading forces, therefore affecting bone’s mechanical and biological properties, increasing its vulnerability to fracture and deformity.

**Figure 4:**
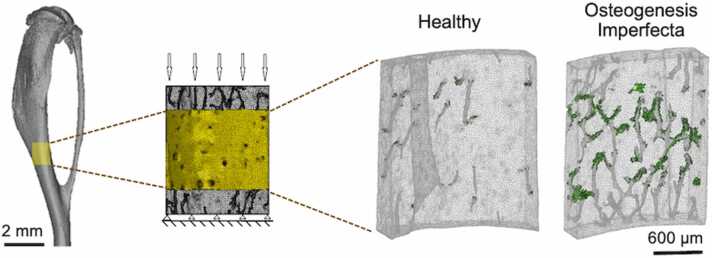
CT reconstruction of a mouse tibia with a posterior midshaft insert scanned with synchrotron CT to show high details of the canal porosity modeled using finite element analysis. The bone blocks were loaded in compression (the volume of interest highlighted in yellow). The two finite element models of healthy and cortical bone blocks shows in green the locations of high risk of fracture initiation when samples are under loading [36], [40]. These locations appear to be around the vascular canals discontinuities and at their intersections. Figure is adapted with permission from Muñoz et al., 2021 [2]. CT, computed tomography.

In classical osteogenesis imperfecta (OI), collagen alterations at the molecular level affect the quality of the bone causing increased fragility [36], [40]. The loss in toughness at the molecular level is due to a decrease in the stabilizing enzymatic crosslinks and an increase in non-enzymatic crosslinks, which leads to smaller and disordered mineralized fibrils that easily stretch and break under load, limiting bone plasticity and favoring crack initiation [36]. Altered fibrils results in disorganized fibers assembled in micro-lamellae. At the tissue level, the high vascular and lacunar porosity reduce the amount of bone material and increases stress concentrations around the voids, favoring the initiation and growth of a crack during loading, increasing the likelihood of fractures (Fig. 4) [36], [40]. This demonstrates how OI modifications of the bone at the molecular level affect overall mechanical integrity of the bone.

**Figure 5:**
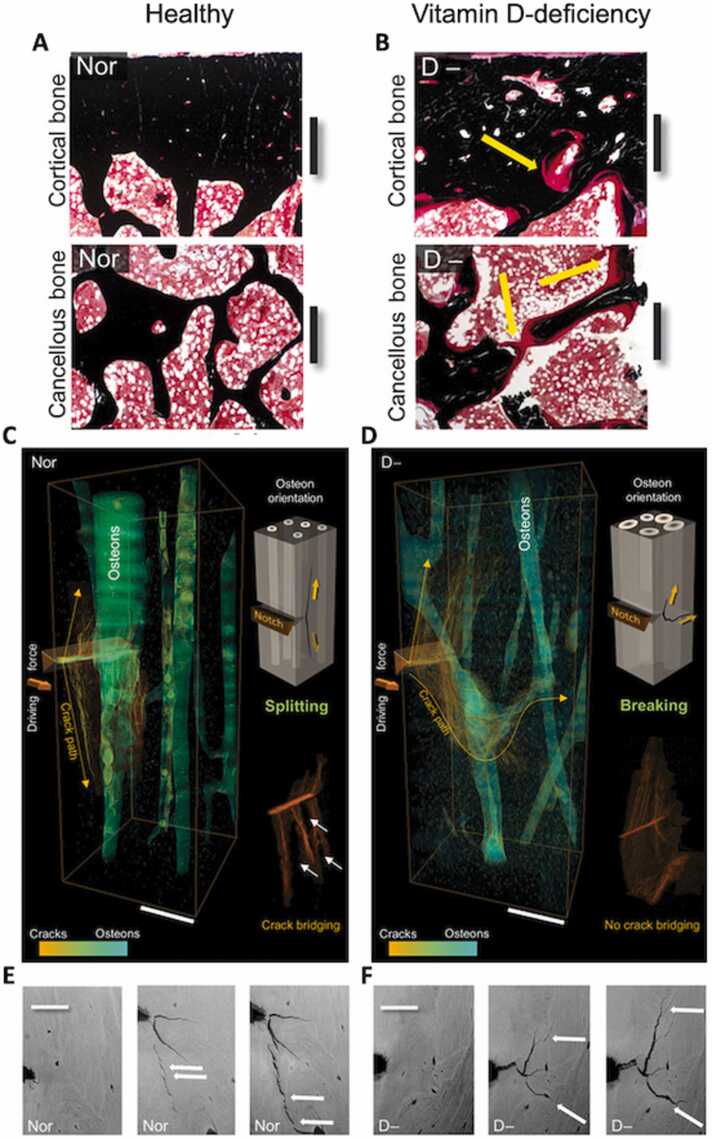
(A) Histology sections of cortical and cancellous with normal osteoid formation and no mineralization defects. Scale bars, 600 µm. (B) Bone sections from vitamin D−deficient subjects reveal an altered bone structure with a thicker layer of unmineralized osteoid coating the surface of mineralized bone as marked by the yellow arrows. Black is mineralized bone tissue; red is bone marrow (von Kossa–stained). Scale bars, 600 µm. (C) 3D reconstruction of the crack path in healthy bone via high-resolution synchrotron radiation micro–computed tomography (SRμCT) exposes crack deflections by splitting along the cement lines surrounding the osteons as well as pronounced crack bridging. Scale bar, 200 µm. (D) In vitamin D–deficient bone, the crack path is much more flat and no crack bridging is visible. Scale bar, 200 µm. (E-F) Environmental SEM images of the crack propagation during fracture toughness for (E) healthy and (F) vitamin D–deficient bone. Uncracked ligament bridges, a major toughening mechanism in bone, are formed in (E) healthy bone but absent in (F) vitamin D–deficient bone. Adapted with permission from Busse et al., 2013 [41]. SEM, scanning electron microscopy.

Similarly, with vitamin-D deficiency, the increased vulnerability to fracture is not simply due to low bone mineral density but rather to alterations in bone composition and structure [41]. Vitamin-D deficient bone has a thick layer of unmineralized osteoid coating the surface of mineralized bone (Fig. 5) [41]. The excess of osteoid prevents bone remodeling because osteoclasts (cells that remove the bone) cannot get through the thick osteoid layer [41]. As a result, the areas of bone hidden underneath the osteoid continue to age and mineralize, becoming increasingly more brittle (Fig. 5) [41]. Thus, vitamin-D deficiency is a complex disease resulting in more than just reduced bone mass.

Laboratory bone analysis of biopsies or ex-vivo animal models of human bone diseases allows examination of structure, composition and mechanics of bone from the molecular to organ level. This allows for observation of the impact of alterations at smaller scales on whole bone strength and toughness, and for demonstration of the efficacy of different treatments. In vivo measurements of bone strength and toughness, however, are more difficult and limited due to the inaccessibility of the material.

## In-vivo screening for bone quantity and quality

### Bone quantity

#### DEXA/DXA

Dual-energy x-ray absorptiometry (DEXA or DXA or bone densitometry), uses a small dose of ionizing radiation to determine the BMD inside the human body in two dimensions (mean areal aBMD, g/cm^2^) [42], [43]. Bone mineral content (BMC, g) can be derived by multiplying the area of the pixel by the aBMD value for that pixel. Summing the total area for all pixels in the region of interest results in total bone area (BA) [44].

**Figure 6:**
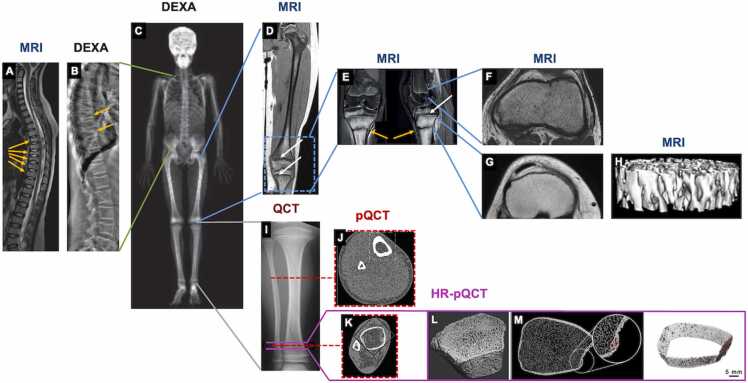
A compilation of current techniques used clinically for the assessment of bone quantity or bone quality parameters in children. (A) Pediatric whole body DEXA scan excluding the head. (B) DEXA image of pediatric lateral spine (C) MRI image of the lateral spine. Yellow arrow indicate vertebral fractures. (D) MRI scan of a whole pediatric femur. (E) MRI scan of the knees. Yellow arrows indicate bone fracture locations and white arrow indicates a growth plate region. (F) Distal femur MRI scan. (G) Proximal tibia MRI scan. (H) A 3D reconstruction of trabecular bone from a proximal tibia 3T MRI scan. (I) CT scout view of tibia. (J-K) pQCT performed in midshaft (J) and distal tibia (K) showing cortical bone and trabecular bone respectively. With pQCT, BMD and microstructural properties of cortical and trabecular bone separately can be determined. (L) 3D reconstruction of distal tibia using HR-pQCT whose structural parameters of both trabecular and cortical bones can be measured. (M) HR-pQCT can distinguish between cortical bone (light grey), intracortical porosity (red), and trabecular bone (dark gray) at each slice of the scan region in the distal tibia so that 3D visualization of the segmented cortical bone (white, transparent) and intracortical porosity (red) shown on the far right can be used for further analysis. Figures are adapted with permission from (A) Bachrach et al., 2007 [49], (B) Binkovitz et al., 2007 [44], (C) Mehany et al., 2021 [54], (D) Carriero et al., 2009 [3], (E) Li et al., 2020 [55], (F) Lerisson et al., 2019 [56], (G) Liu et al., 2018 [57], (H) Abdalrahaman et al., 2015 [58], (I-L) Adams et al., 2014 [59], (M) Burghardt et al., 2010 [60]. BMD, bone mineral density; CT, computed tomography; DEXA, dual-energy x-ray absorptiometry; MRI, magnetic resonance imaging.

DEXA scans include both trabecular and cortical bone in an indistinguishable manner (Fig. 6A and B). Compared to conventional radiographs, DEXA scans have reduced resolution, but also reduced radiation exposure [45], [46], [47], [48], [49]. Bone mass is interpreted either in terms of T-score in adults [50], [51] or in terms of Z-score in comparison to the average bone mass of same age and sex population. Z-score is used in skeletally immature subjects. A correct Z-score must be adjusted for age, gender, body size, pubertal status and if possible, ethnicity according to the International Society for Clinical Densitometry [52]. Under the age of 5, DEXA is not useful because there is no age-matched reference data for interpretation [53].

A diagnosis of osteopenia in children is based mainly on a low aBMD and at least one low trauma fragility fracture (Fig. 6B). DEXA has not been found to be reliable when trying to use BMD Z-scores for prediction of fractures in children. Using a 2-dimensional image from DEXA has disadvantages. The lack of depth information on bone microarchitecture and volumetric bone density makes DEXA scans particularly difficult to interpret. This is especially true for profound osteopenia, as seen in the severe form of OI, type III OI. It may reflect the short body stature of the child and not necessarily “altered bone” [53]. In children and adolescents with vitamin-D deficiency, despite the low aBMD, no significant association was found between vitamin-D levels and DEXA parameters of bone density [61], [62]. Routine vitamin-D testing may be a more helpful indicator of bone health than a DEXA study in young patients with fractures [61], [62], [63], [64].

Despite its limitations, DEXA remains the sole practical tool for measuring bone mass in children and is used to obtain a measurable look into the course of disease and efficacy of treatments in children with extreme bone fragility [65]. Fracture rate, [66] and the effect of recombinant growth hormone treatment [67] and/or bisphosphonates [42], [68], [69] have been examined using DEXA aBMD or apparent BMD in the lumbar spine. The vertebrae in children with OI, however, are difficult structures for DEXA mapping, due to the intense osteopenia at the edges of the bones [70]. Furthermore, DEXA-based fracture prediction tools used in adults, such as FRAX thresholds (10-year fracture probabilities) are not valid in children, [51] and vertebral fracture assessment (VFA) can be misleading in cases of physiological reductions in vertebral height or in conditions such as Scheurmann’s disease [71].

#### Quantitative computed tomography (QCT), peripheral pQCT and high resolution peripheral HR-pQCT

Quantitative computed tomography (QCT) overcomes the 2-dimensional limitations of DEXA by offering a 3D scan modality that allows the user to quantify the true physical volumetric BMD (vBMD) (g/cm^3^ or mg/cm^3^) and BMC (g). QCT usually refers to whole body CT, but there are also dedicated techniques such as peripheral pQCT and high-resolution peripheral HR-pQRT that offer targeted images (Fig. 6I-M) [59]. These three techniques can with sufficient accuracy, separate and describe the trabecular and cortical bone compartments, a distinction DEXA is unable to attain [59]. The risk of developing radiation-related cancer from CT exposure is considerably higher in young children than in adults exposed to the same CT scan multiple times, making CT unfeasible for routine use in the pediatric population [72]. pQTC is associated with lower radiation compared to conventional QCT. Assessment of peripheral sites with pQCT, has advantages over DEXA scans in the pediatric population with spinal deformities, contractures or metallic implants despite its higher dose of radiation [73]. pQCT, however, is highly sensitive to movement, making this a difficult study to obtain on young children [73]. For this reason and the higher level of radiation compared to DEXA, pQCT has not been widely researched or adapted clinically.

Reconstructed CT images result in grayscale values, which are representative of mineralization. Calibration of CT to BMD values is made possible by the use of a phantom (object) made of hydroxyapatite with a known density [59]. While water and bone are considered the main constituents during scanning, CT value for water being 0 Hounsfield units (HU), fat is as important of a component, especially in growing children [59], [74]. Fat has a lower density than water resulting in CT values less than 0 HU, which by default artificially lowers the overall vBMD. In growing children the red hematopoietic marrow is gradually changing into yellow marrow resembling fat, so that the vBMD values would be continually changing, adding another level of complexity especially in longitudinal studies [59], [75].

### Bone quality

#### Quantitative computed tomography (QCT), peripheral pQCT and high resolution peripheral HR-pQCT

Quantitative parameters of bone quality for cortical and trabecular bone compartments can be calculated with QCT, p-QCT and HR-pQCT (Fig. 6I-M) [59], [60]. Geometrical measurements such as bone cross-sectional area (CSA) and cortical thickness, area and volume as well as biomechanical parameters, such as cross-sectional moment of inertia (CSMI) as a measure of bone strength can be calculated with QCT, p-QCT and HR-pQCT [59]. HR-pQCT is also used to assess cortical and trabecular macro- and microarchitecture, particularly at the distal radius and tibia (Fig. 6L-M). There is evidence that children and adolescents with a distal forearm fracture due to mild, but not moderate, trauma have thinner bone cortices and deficits in trabecular bone microstructure, [76] which can in turn compromise their bone strength and resistance to fracture. More recently, a significant positive correlation between vitamin-D levels and HR-pQCT derived bone quality parameters for trabecular bone volume fraction (BV/TV) and thickness (TbTh) and negative correlation with trabecular spacing (TbSp) in a large cohort of girls and boys [77].

#### Magnetic resonance imaging (MRI) and spectroscopy (MRS)

Magnetic resonance imaging (MRI) is a powerful non-ionizing, non-invasive and painless modality that produces 3D imaging. MRI is a very useful clinical tool because it can image both soft and hard tissues simultaneously (Fig. 6C-G). It uses a magnetic field to create a detailed cross-sectional image of both cortical and trabecular bone, [3] and allows for the analysis of bone micro-architecture at high detail (Fig. 6F-H). This micro-architecture includes the assessment of apparent bone volume-to-total volume ratio, apparent trabecular number (appTbN), thickness (appTbTh), and separation in trabecular bone [58], [78]. MRI is not available for routine use due to its high costs and need for general anesthesia in the young population. MRI has not yet been utilized to assess health of long bones in children or young adults with OI, [79], [80] but has been utilized for children with osteosarcoma, [81], [82] diabetes, [58], [78] and cerebral palsy [3], [83]. In particular, a deficit in trabecular bone microarchitecture (appBV/TV and appTbN) was observed in children with type 1 diabetes (T1D) [58], [78]. However, no association was found between trabecular features and fractures in this population.

Bone microenvironment consisting of bone marrow fat content and composition can be estimated via magnetic resonance spectroscopy (MRS) [84]. MRS performed on the vertebrae of children with T1D to assess the lipid-to-water ratio and percentage fat fraction, [85] correlates positively with trabecular spacing [58] and inversely with trabecular number (appTbN). This reinforces the hypothesis that the observed skeletal deficit in T1D may have its origins in a shift of mesenchymal stem cell differentiation toward adipogenesis rather than osteogenesis [78].

#### Quantitative ultrasound

Unlike DEXA and CT, ultrasound is a non-invasive imaging modality with no associated radiation offering several advantages including affordability, portability, and easy isolation of a specific anatomical location. Although its utility can be user dependent, its advantages make this a favorable assessment tool for bone quality in children and adolescents [86].

Ultrasound waves interact with bone in a very different way compared to ionizing radiation and can provide information about bone properties including tissue density, elasticity and architecture [86], [87], [88], [89]. In trabecular bone, wave attenuation occurs in a scattering fashion causing the energy to dissipate along the complex architecture of the tissue [90]. In cortical bone, acoustic energy is predominantly absorbed and subsequently converted to thermal energy [91]. Thus, quantitative results that can be derived from ultrasound imaging include: amplitude-independent velocity, speed of sound (SoS), amplitude-dependent SoS (AD-SoS), [91], [92] and broadband ultrasound attenuation (BUA) from quantitative ultrasound devices [54]. Bone transmission time can be calculated, which characterizes bone properties independent of the effect of surrounding soft tissue [90].

**Figure 7:**
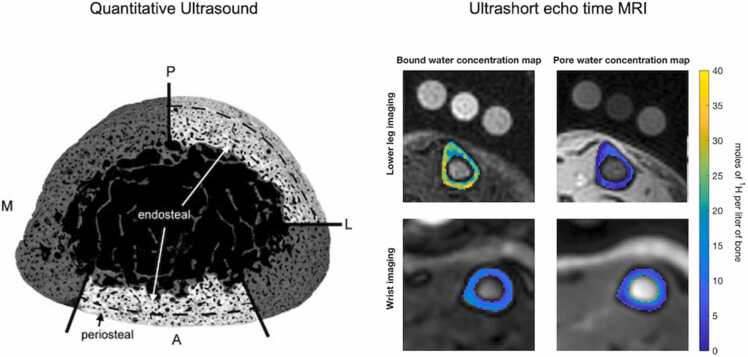
Future directions (A) Acoustic impedance image obtained from quantitative ultrasound (QUS) of an excised radius sample from an adult human. QUS can be used to quantitatively assess bone material properties as it can extract structural parameters of bone with high accuracy. M = medial, P = posterior, L = lateral, A = anterior. (B) Ultrashort echo time (UET) MRI-derived concentration maps for bound water and pore water from 2D scans of the tibia mid-diaphysis (top) and 3D scans of the distal radius (bottom). QUS and UET MRI images adapted with permission from Raum et al., 2005 [93], and Nyman et al., 2023 [98], respectively.

Ultrasound generally uses axial, biaxial or through-transmission modes. In biaxial mode, ultrasound or bidirectional axial transmission (BDAT) ultrasound, the velocity of the first arriving signal has been directly linked to cortical tissue stiffness, cortical thickness and cortical porosity (Fig. 7A) [93]. For this reason, BDAT ultrasound could be used to monitor treatment of children and young adults with bone diseases. When BDAT ultrasound was used in 4-year-old children with X-linked hypophosphatemic rickets, lower velocity signals were observed compared to age-matched controls [94]. These lower velocity signals are directly linked to cortical tissue stiffness, porosity and thickness. Because of the high sensitivity and specificity of ultrasound in detection of fractures in children, this imaging technique could be used for evaluation and assessment of fractures in the pediatric population [95]. However, further research is needed to determine whether such methodology is advantageous over DEXA or pQCT to assess bone health [96], [97].

#### Biochemical markers

Bone is a highly metabolic system, with a fine balance between formation and resorption. During growth, bone formation and modeling are predominant over resorption. Nevertheless, in disease this balance can be impaired, leading to changes in bone quality and quantity. That is why laboratory tests are conducted frequently in children showing signs of bone fragility. Such biomarkers are influenced by the children’s age, gender and pubertal stage, and are essential in monitoring treatment therapies because changes in bone turnover markers (BTMs) in response to treatment are much more rapid and dynamic than changes in BMD [99].

Current non-invasive bone health screening includes laboratory testing of blood and urine. Blood samples can be collected from children preferably at a time that coincides with the clinic visit, and comprehensive metabolic analysis can be conducted, including calcium and phosphate for proper bone mineralization, magnesium to check for impurity in bone, albumin, and alkaline phosphatase levels for bone formation [45]. Parathyroid hormone (PTH) is also measured to assess the calcium level in the blood, which is a reflection of health problems in the bones [100]. Finally, 25-hydroxyvitamin D assay is used to determine level of vitamin-D in the body. In the urine, other laboratory tests can be routinely conducted, such as pyridinoline (PD) and deoxypyridinoline/creatinine ratio (DPD/crea) for bone resorption, and calcium/creatinine levels for osteopenia [68], [101]. Controversy exists regarding these two values. They do not reliably predict aBMD in children, both in those with healthy bone [102] and with those with metabolic disorders [101]. Finally, hormonal biomarkers, such as Serum PFAS and Urinary Phthalate, and bone turnover markers are tested in children and adolescents with known disrupted hormonal signaling pathways affecting bone homeostasis [103], [104], [105].

## Future directions for fracture risk prediction

Currently, DEXA is used to provide information about relative fracture risk to determine whether treatment is required, and/or to assess efficacy of treatments. However, DEXA is not highly accurate in determining BMD, particularly in children. Because bone quantity correlates poorly with bone toughness, DEXA poorly predicts fracture risk. For this reason, aBMD DEXA is used in conjunction with FRAX threshold - with limited success - in predicting fracture risk in adults, [106], [107], [108] and is not valid for assessment in children [51]. Future bone health screenings will be needed to determine bone quality (i.e., structure, composition, microdamage, modeling and remodeling) parameters other than microarchitecture in vivo and non-invasively, to effectively estimate bone fracture toughness (i.e., fracture risk). Future bone quality assessment relies on the development of new non-invasive approaches that would have screening and diagnostic potential in the clinical setting. Techniques currently being researched for assessment of bone quality and prediction of bone fragility include: (1) Raman spectroscopy to analyze bone composition, (2) UTE-MRI to investigate bone pore and bound water, (3) ultrasound to determine bone quality properties; (4) genomic advancement to establish relevant RNA biomarkers. The socio-economic benefit of this research would help not only the pediatric population suffering from bone fragility, but would be impactful worldwide for those with fragility fractures. In the US alone, fragility fractures impact 1.5 million people each year [109].

### Raman spectroscopy

Since its first development in 2005, [110] the spatially offset Raman spectroscopy (SORS) [111] technique has seen many applications for non-invasive determination of bone quality properties in vivo. However, its implementation clinically has yet to follow. Particularly, in 2014, Buckley and colleagues demonstrated the relevance of SORS for assessing bone composition and suggested its utilization in determining bone compositional abnormalities in osteoporosis, osteoarthritis and osteogenesis imperfecta in clinical settings, [112] as previously identified in excised bones [111], [113]. In their studies, Buckley and colleagues performed different multivariate analyses combined with SORS to extract the compositional spectrum of in vivo transcutaneous human bone tissue. They observed that SORS can give access to the chemical information of bone tissue on both organic and inorganic components that contribute to bone mechanical properties and describes bone quality. Ideally experts would be able to use SORS to predict whether or not a patient will sustain a fragility fracture [114], [115], [116]. Recently, Unal and colleagues [116] found a correlation between bone resistance to crack initiation and a combination variables including age, aBMD and Raman (probe) value. While this does not fully explain bone resistance to fracture, it represents a first step towards a new approach for predicting fracture risk in a clinical setting [116]. More preclinical studies are needed to show which bone components are associated with fractures in trabecular and cortical bone of children with different diseases. Raman spectroscopy is a very promising technique for future non-invasive assessment of bone health.

### MRI

Ultrashort echo time (UET) MRI-derived measurements of bound and pore water concentrations in people with fragility fracture could be a promising predictor of fracture risk and therapy efficacy (Fig. 7B) [98]. In a recent study, concentrations of bound water in osteoporotic patients with fragility fractures were lower than in the control group after 6 months of therapy, whereas concentrations of pore water showed no difference between groups [98]. These markers play a crucial role in bone as pore water is an indicator of cortical tissue porosity, while bound water can be a marker of tissue hydration state [117]. Both these properties are key players in bone toughness. More preclinical studies aimed at analyzing the water content of bone in vivo are needed to better evaluate the utility of this imaging modality.

### Ultrasound

A modern ultrasound axial transmission (AT) system including a custom-made probe, driving electronics and a human machine interface set-up was used to predict cortical thickness and cortical porosity of cadaveric human tibia with no muscles and skin attached [118]. Both estimations for thickness and porosity were successfully validated by high-resolution micro-computed tomography (μCT). While the translation to in vivo of this technique is not direct, studies using ultrasound technology based assessment for bone fracture risk should be pursued due to their low cost and maintenance, and lack of radiation risk.

### RNA biomarkers

More recent genomic work uses RNA biomarkers as a potential tool for identification of certain bone diseases. MicroRNAs (miRNAs) are among the non-coding RNAs that hold crucial epigenetic regulator roles in many bone diseases and can be reliably detected in blood samples [119], [120], [121]. MiRNAs are crucial factors in bone development, growth and regeneration, which is why they tend to be very compelling future biomarkers for bone quality. Currently, one example of such biomarker is miRNA-21, which when coupled with nuclease digestion, can help in identifying osteosarcoma in children and adolescents [122].

Similarly, circular RNAs (circRNAs) play a vital role in cellular activity and bone metabolism [123], [124], [125], [126] and are promising biomarkers in diagnosis, prognosis and treatment methods for bone hemostasis disorders such as osteoporosis, Paget’s disease, rickets and osteopetrosis [127]. Further research and validation is needed to determine the potential of such biomarkers [128]. In the future, circRNAs might be beneficial for fracture risk assessment in patients with metabolic bone diseases [129].

## Conclusion

Healthy bone is strong and tough. When clinically evaluating for bone fragility disorders, we must consider changes in bone quality and not just quantity because bone’s ability to resist fracture depends highly on its structure and composition. Thus, clinical fracture risk should be a function of both bone structure and composition, rather than BMD alone. For this to be successfully achieved, there is still a critical need for (1) pre-clinical testing analyzing structure, composition and toughness to better understand bone fragility and assess treatment success, and of (2) clinical tools that can efficiently predict fracture risk considering bone quality properties.

## Author contributions

**Alessandra Carriero:** Conceptualization, Funding acquisition, Investigation, Methodology, Project administration, Resources, Supervision, Visualization, Writing – original draft, Writing – review & editing. **Anxhela Docaj:** Data curation, Investigation, Methodology, Visualization, Writing – original draft, Writing – review & editing.

## Declarations of competing interests

The authors declare the following financial interests/personal relationships which may be considered as potential competing interests: Alessandra Carriero reports financial support was provided by National Science Foundation. Alessandra Carriero reports financial support was provided by Human Frontier Science Program. Alessandra Carriero reports a relationship with Fellows of Politecnico di Milano, US that includes: board membership. Alessandra Carriero reports a relationship with the American Society of Bone and Mineral Research that includes: member of the Finance Committee. If there are other authors, they declare that they have no known competing financial interests or personal relationships that could have appeared to influence the work reported in this paper.
